# A Non-Stochastic Special Model of Risk Based on Radon Transform

**DOI:** 10.3390/e26110913

**Published:** 2024-10-28

**Authors:** Marcin Makowski, Edward W. Piotrowski

**Affiliations:** Faculty of Physics, Department of Mathematical Methods in Physics, University of Białystok, ul. Ciołkowskiego 1L, 15-245 Białystok, Poland; e.piotrowski@uwb.edu.pl

**Keywords:** risk, complex system, error, Radon transform, financial instrument, market, uncertainty, information

## Abstract

The concept of risk is fundamental in various scientific fields, including physics, biology and engineering, and is crucial for the study of complex systems, especially financial markets. In our research, we introduce a novel risk model that has a natural transactional–financial interpretation. In our approach, the risk of holding a financial instrument is related to the measure of the possibility of its loss. In this context, a financial instrument is riskier the more opportunities there are to dispose of it, i.e., to sell it. We present a model of risk understood in this way, introducing, in particular, the concept of financial time and a financial frame of reference, which allows for associating risk with the subjective perception of the observer. The presented approach does not rely on statistical assumptions and is based on the transactional interpretation of models. To measure risk, we propose using the Radon transform. The financial concept of risk is closely related to the concepts of uncertainty, entropy, information, and error in physics. Therefore, the well-established algorithmic aspects of the computed tomography method can be effectively applied to the broader field of uncertainty analysis, which is one of the foundational elements of experimental physics.

## 1. Introduction

Risk is one of the key elements analyzed in the context of the functioning of financial markets, which are well-defined complex systems. These markets, being under constant observation, provide a vast amount of data, which fosters the development and testing of scientific theories [1,2]. Therefore, although risk has an interdisciplinary nature, it is worth studying in the financial context, as the conclusions from such analyses can also be useful in research on complex systems in general.

The literature is dominated by an approach [3,4] that focuses on volatility as the main measure of risk. Volatility is typically defined as the standard deviation of price changes over time, which has become the foundation of risk research since the introduction of Harry Markowitz’s Modern Portfolio Theory (see, e.g., [5]). The importance of volatility is evidenced by the multitude of statistical models developed for financial markets, including the Black–Scholes model [6]. Greater volatility is usually associated with higher risk and instability. However, recent studies suggest that both high and low volatility can lead to instability in financial markets [7]. This shows that, although the concept of volatility has a long history and forms the basis of financial research, there are still aspects of this phenomenon that are not fully understood.

Interestingly, risk does not have a single, coherent definition, yet most existing models and measures of risk are based on a statistical approach, which traces its origins to the work of Louis Bachelier, who was the first to observe that the logarithms of prices follow a specific one-dimensional Brownian motion. This traditional approach often fails to predict financial crises and fully describe the dynamics of financial markets. Hence, there is a need to explore new risk models, including non-statistical ones, and those that emphasize that risk is fundamentally subjective. A source of inspiration here can be the physical models developed within the framework of so-called econophysics [8,9,10,11,12,13,14,15], a relatively new field that has evolved over recent decades and employs research methods from mathematics, physics, and economics. This approach seems particularly relevant due to the interdisciplinary nature of risk and its connection with the concept of information (see, e.g., [14]), which may lead to new insights in fields of knowledge often far removed from finance.

In this article, we present a new model of financial risk and a method for its measurement using the Radon transform. The next section is devoted to explaining the concepts behind the proposed approach. In Section 3, we provide a brief introduction to the geometric theory of the market, which forms the basis for our subsequent analysis. Section 4, Section 5, Section 6 and Section 7 develop the theoretical framework of the proposed model, including the frame of reference, the introduction of the concept of reference time, and the definition of a financial instrument’s graph. In Section 8, we differentiate so-called simple instruments from the broader set of financial instruments and provide examples. In our model, these instruments carry the lowest risk and play a crucial role in the risk measurement method. In Section 9, we review the basic properties of the Dirac delta function, which helps us, in Section 10, to represent the graph of a financial instrument and define its Radon transform. In Section 11, we introduce the concept of the reachability of a financial instrument and use it to define its risk. Section 12 discusses the interpretation of reachability as a measure of risk. General remarks regarding the extension of the model and its significance for the advancement of risk research are presented in the final section, Conclusions.

## 2. The Idea behind Our Approach to Financial Instrument Risk Modeling

It is a common belief that the greater the number of ways a good can be lost, the riskier it is. This idea stems from the simple observation that each potential path of loss increases the uncertainty of maintaining possession of the good in the future. Such an assessment should also be shared by the future purchaser of this good.

In the case of financial instruments, excluding the effects of crimes, loss can occur through sale. Of course, making transactions in the market is something natural. However, it is at the moment when we make the decision to sell or buy that risk ceases to be merely theoretical and becomes real. For example, when you decide to sell shares, you risk that the price may fall right after the sale. However, there is also the risk that the price could rise, which means bearing the cost of a missed opportunity. An investor does not only play against the market, but also has to contend with their own emotions. The more such crossroads, the greater the risk of making poor decisions.

It is worth noting that the terms ‘sale’ or ‘purchase’ refer only to the direction of the flow of a particular good, and in the context of assessing the frequency of transactions, the positions of seller and buyer appear to be fully symmetrical. The buyer, in fact, exchanges one good (e.g., money) for another that is currently desired by them. Therefore, the measure of the possibility of selling a financial instrument is equal to the measure of the possibility of buying it. As mentioned above, risk may be associated with the possibility of buying or selling goods. In this context, an object of transaction, such as a financial instrument, becomes increasingly risky as the number of opportunities to purchase or sell it rises.

Below, we propose a novel method, not previously discussed in the literature, for determining the risk associated with a financial instrument, based on the measure of opportunities to buy or sell it. These opportunities may fluctuate due to various factors such as a reassessment of the expected profits from the instrument. The link between market risk and transactions is standard because they are the generators of prices in the market. Note that almost all risk measures take into account price fluctuations, with the main difference between them being that they evaluate it in relation to other market factors. The popular standard deviation measures how much asset prices can fluctuate around the average. In this article, we propose linking risk to the subjective perception of the observer, specifically the measure of potential subjective transactions. This is not a new concept, as there has long been a discussion in the literature about the reasons for the different assessments of the same risk by different market entities. Often the role of psychological and social factors is emphasized here [16]. One attempt to capture this phenomenon is prospect theory, introduced by Daniel Kahneman and Amos Tversky in 1979 [17,18,19]. This theory explains how people decide to take risks in situations where they can win or lose, and these choices are often contrary to classical utility theory. Our approach is based on introducing a frame of reference from which the investor assesses the risk of a financial instrument. We consider the potential transactions that an observer of a given financial instrument could have executed with respect to that instrument. This is a particular type of transaction, taking into account the individual perspective of the observer. Thus, we distinguish this type of transaction among all others that affect the shape of the financial instrument’s graph. It is not about the actual transactions made in the past, but rather about all the possible moments when the transaction could have been realized—that is, all those situations when the market participant faced the dilemma of whether to buy, sell, or not to take any action.

The process of buying or selling a financial instrument here involves replacing the instrument in question with another of the lowest possible risk. It is important to note that this behavior is commonly observed in stock exchanges, where the least risky asset is typically considered to be money, although it depends on the reference system, non-risk instruments may be different here. When an investor sells a risky financial instrument, they are effectively exchanging it for money. Subsequently, they may use this money to purchase another risky instrument. Typically, there are various instruments, not only money, that investors use to store their capital between transactions involving higher-risk financial instruments.

The proposed measure of risk is based on a special type of financial instrument that possesses the lowest risk within a specific frame of reference, known as simple instruments. The representations of such instruments are segments of straight lines. The moment of possible exchange of financial instruments is graphically interpreted as the point of intersection of the graphs of both financial instruments. This is the point at which a simple, less risky instrument has the same price—or, more precisely, the same rate of return—as the instrument in question. The owner of this instrument is thus confronted with a dilemma: whether to replace this instrument with a less risky one or not. The greater the number of such dilemmas, the greater the risk associated with the instrument. As we can infer, this dependency is related to the shape of the financial instrument’s graph. A randomly drawn straight line is more likely to intersect multiple times with a jagged section of the graph, which has numerous twists and turns, than with a smoothed section that has a regular course. It is worth remembering that in the statistical interpretation of risk as the dispersion of a random variable, we assign a higher risk to variables with greater variance.

The advantage of the proposed measure of risk is its direct connection with possible subjective buy and sell transactions, namely, the exchange of instruments, on a given market. Transactions, their quantity, and availability on a given market have a direct impact on price fluctuations, and thus, on the risk of choosing the wrong price. A significant benefit is also the fact that the risk measure is a natural equivalent of the Radon transform. This transformation has led to a revolution in the field of medical imaging. Furthermore, it is used in astronomy, optics, geophysics, and in the analysis of financial markets, particularly in the study of speculative bubbles [20]. It might also help us to think about financial instruments in a different way. Risk is such an important part of financial instruments that it is worth examining using various mathematical methods. This approach could lead to better, more accurate models of the dynamics of financial markets and to the discovery of fundamental laws of the financial world. Additionally, the results could inspire new research in other fields of science, based on broadly understood studies of complex systems.

## 3. Portfolios

Economists often engage in analyzing the relationships between various quantities. In the context of prices and asset values, the proportions between them provide crucial information. When ownership shares in assets become available in fractional parts, absolute measures lose significance, and the common tendency of investors to diversify risk makes such a form of asset ownership standard. The market is a space where the free exchange of goods, services, and capital takes place. To better understand the mechanisms governing this process, we seek models that capture its key features.

Let us assume that the objects of interest for traders (financial instruments available on the market) span the (n+1)-dimensional vector space *I* over R. The elements of the space *I* are called *baskets*. The term evokes a justified association with baskets, which are the result of an entire day of shopping, filled with various items, including smaller baskets (addition). Each element of the basket can appear in greater or lesser quantities (multiplication by a positive number). They can contain, in addition to assets, liabilities as well (multiplication by −1). Considering liabilities or fractional parts of indivisible items is fully justified, as our baskets represent ownership rights (and assumed liabilities), which can formally encompass any fractional parts of unitary objects through appropriate intermediary agreements. The mentioned algebraic operations possess the appropriate properties (such as, among others, associativity and commutativity), which fully characterize them as a vector space.

Let us choose the basis $\left\{v_{0},v_{1},v_{2},\ldots ,v_{n}\right\}$ in *I*, where the element $v_{i}\in I$ denotes the *i*-th financial instrument. For any p∈I, we have its unique representation:

$$p=\sum_{i=0}^{n}p_{i}v_{i}.$$

The coefficient $p_{i}\in R$ is called the *i*-th coordinate of the basket. A portfolio is defined as an equivalence class on I∖{0} (non-empty baskets). Two baskets $p^{\prime }$ and $p^{\prime \prime }$ are equivalent if and only if there exists λ∈R∖{0} such that $p^{\prime }=\lambda p^{\prime \prime }$.

In this way, we identify baskets with the same assets in the same proportions. This is reasonable because the properties of such baskets (such as the rate of return or risk) that we are interested in depend only on their composition and proportions, and not on the value of individual elements of the basket [21].

If for a given portfolio we have $p_{i}\neq 0$, then there exists such a basket representing this portfolio that it contains exactly a unit of asset $v_{i}$. The coordinates of this basket,

$$p=(p_{0},\ldots ,p_{i-1},1,p_{i+1},\ldots ,p_{n})\;,$$

are called inhomogeneous coordinates of the portfolio *p* with respect to the *i*-th asset. The market price *U* with respect to the *i*-th good is any linear mapping $U(v_{i},\cdot ):I\to R$. The mapping *U* assigns to each basket *p* its current value, expressed in units of the good $v_{i}$. More interesting details on the geometric structure of the market, in particular its relationship to projective geometry, can be found in [21]. We will not quote them here. In the following paragraphs, we will only introduce concepts relevant to our further considerations, such as *frame of reference* and *simple instrument*. They will be used to describe the geometric interpretation of the risk of a financial instrument.

## 4. The Frame of Reference

The valuation of a financial instrument is made in relation to a certain amount of another distinguished financial instrument, which we will call *money* and denote by $v_{0}$. Money is commonly accepted as the medium of exchange and unit of account; it serves as the universal measure of value in a given market. As such, money is a financial instrument that enables assets to be valued uniformly and consistently, facilitating comparisons of their value across different markets, which may use different forms of money.

In the set *I*, let us distinguish another financial instrument $v_{1}$, which we will call a *benchmark* instrument. The pair **(money, benchmark)** is defined as **the frame of reference** for the market.

Following the example of physical systems, the introduction of the concept of a frame of reference in finance is a response to the relative nature of quantities characterizing a financial instrument, including its associated risks. Risk is inherently subjective: its perception can vary widely depending on personal experience, knowledge, values, and emotions. What one person considers risky, another might deem acceptable or even safe. For instance, someone who has faced negative outcomes from risk-taking in the past might view similar future situations as more perilous.

Risk assessment is often context-specific. A financial investment might be seen as riskier in an unstable economic environment than in a stable one. Moreover, assets—financial or otherwise—can be valued in various currencies and markets. For example, the perceived risk of a particular good can greatly depend on the currency in which it is priced. An example could be an investment in oil, whose contracts are settled in U.S. dollars. Observing these contracts in a different currency can significantly influence the perception of risk associated with these financial instruments, stemming from the exchange rate relationship of the dollar to other currencies.

Introducing a frame of reference allows for the standardization of risk measurement, thereby facilitating easier comparison of risks across different options, scenarios, or projects. Without such a common baseline, comparing risks would be significantly more challenging, and the conclusions drawn from such comparisons would lack validity, as each situation would be assessed against a unique set of criteria.

The benchmark instrument and the frame of reference are not merely abstract concepts. According to research conducted by the creators of prospect theory [17], people evaluate the alternatives available to them based on a certain reference point, which is determined by their current wealth, past experiences, and other factors. For some, the benchmark may be government bonds, while for others, it may be the price of gold. Our model is general and does not propose a specific interpretation here. The choice of the frame of reference is a subjective decision made by the market analyst.

## 5. The Reference Time

It is not clear whether astronomical time is an appropriate tool for measuring the passage of time in finance. This doubt stems from the fact that market transactions seem to be concluded at random moments and with random intensity. Moreover, business cycles (intervals between successive crises) do not show the regularity that accompanies biological or chemical cycles. This may prompt the introduction of the concept of reference time.

Let us denote by $U_{t}\left(v_{0},\theta \right)$ the price (at time *t*) of a unit of good θ expressed in units of $v_{0}$ (money). Since $U_{t}\left(v_{0},v_{0}\right)=1$ (for any *t*) we are able to conclude that the market rate of the goods $v_{0}$, $v_{1}$, $v_{A}$ is uniquely defined by the point of the 2-dimensional projective space with homogeneous coordinates

$$(1,\;U_{t}\left(v_{0},v_{1}\right),\;U_{t}\left(v_{0},v_{A}\right)\;)\;.$$

Moving on to the logarithms of prices, let us introduce the notation

(1) $$\begin{array}{cc} \ln U_{t}\left(v_{0},v_{1}\right)-\ln U_{t_{0}}\left(v_{0},v_{1}\right) & :=x(t),\\ \ln U_{t}\left(v_{0},v_{A}\right)-\ln U_{t_{0}}\left(v_{0},v_{A}\right) & :=y_{A}\left(t\right)\;, \end{array}$$

where $t_{0}$ is an arbitrarily chosen but fixed moment of time. The above formulas represent logarithmic rates of return.

Let *t* be astronomical time, measured in any units. We call the function r(t) the instantaneous rate of growth of the benchmark instrument relative to money:

(2) $$r\left(t\right):=\frac{1}{U_{t}\left(v_{0},v_{1}\right)}\frac{d\;U_{t}\left(v_{0},v_{1}\right)}{dt}=\frac{d}{dt}\;\ln U_{t}\left(v_{0},v_{1}\right)\;,$$

where $U_{t}\left(v_{0},v_{1}\right)$ is the price at time *t* of the benchmark instrument (in monetary units).

By ***reference time*** τ(t) we mean the definite integral of the instantaneous rate of growth of the benchmark instrument r(t), taken at some initial moment $t_{0}$ determining the beginning of the reference time:

(3) $$\tau \left(t\right):=\int_{t_{0}}^{t}r\left(t^{\prime }\right)dt^{\prime }\;.$$

It follows from the above definitions that the reference time depends on the choice of frame of reference—the choice of money and benchmark instrument. Note that from Equations (1)–(3) it follows that

$$\tau \left(t\right)=\ln U_{t}\left(v_{0},v_{1}\right)-\ln U_{t_{0}}\left(v_{0},v_{1}\right):=x\left(t\right).$$

It is important to note that the reference time, which represents the proportion of the benchmark instrument’s prices, is a dimensionless quantity. Thus, it is an atypical measure of time but it accurately reflects economic realities. The concept is modeled after the way time is measured in thermodynamic problems [22], where it often refers to the time during which a characteristic process occurs, such as the time needed for a system to reach equilibrium. Time is then measured not in absolute units (such as seconds), but in relation to how quickly a specific process occurs, for example, energy dissipation or temperature change. This allows us to compare different processes more universally by analyzing their mechanisms rather than the direct passage of time. Similarly, we measure time by the growth of a benchmark instrument. Using this convention, we might say, for example, that it lasted until the price of a good became 1.5 times higher, or that one should buy when the stock price drops by 60%. Such a defined time cannot be expressed in astronomical terms; it is a dimensionless quantity. This is important because it permits universal comparisons and scaling across diverse systems or frames of reference without the need for unit conversions, thereby facilitating more comprehensive and consistent scientific and engineering analyses.

Some comments regarding reference time can also be found in the conclusions of this paper.

## 6. The Graph of a Financial Instrument

We can look at the graph of a financial instrument as a curve.

**Definition** **1.** *The curve*

(4) $$\gamma_{A}\left(t\right):=\left\{\left(\;x\left(t\right),\;y_{A}\left(t\right)\;\right)\;\in R^{2}\;|\;t\in \left(t_{0},t_{n}\right]\right\}$$

*is called the graph of the financial instrument parametrized by astronomical time t.*

The restriction of *t* to the interval $\left(t_{0},t_{n}\right]$ is only practical. This interval can be arbitrarily long. In fact, the available historical data are limited to the period during which a specific financial instrument was actively observed and traded. This means that we observe a financial instrument over some finite reference time period. The end of this period is at $t_{n}$. We omit the initial reference time $t_{0}$ as it is not relevant; in any frame of reference, we have $x\left(t_{0}\right)=y\left(t_{0}\right)=0$. The risk measure proposed below is based on the shape of the curve, rather than its position in space. Consequently, curves of the same shape are identified with each other, as will be discussed in the following remark.

**Remark** **1.** *Two curves $\gamma_{A}\left(t\right)$ and $\gamma_{A}^{\prime }\left(t\right)=\left\{\left(\;x^{\prime }\left(t\right),\;y_{A}^{\prime }\left(t\right)\;\right)\;\in R^{2}\;|\;t\in \left(t_{0},t_{n}\right]\right\}$ are said to be equivalent if there exists a vector $\left(a,b\right)\in R^{2}$ such that*

$$\left(\;x^{\prime }\left(t\right),\;y_{A}^{\prime }\left(t\right)\;\right)=\left(x\left(t\right)+a,y_{A}\left(t\right)+b\right)\;\mathrm{for}\;\mathrm{all}\;t\in \left(t_{0},t_{n}\right]\;.$$

*This relation is an equivalence relation. All curves that are translations of each other form an equivalence class. This identification of curves is justified because financial instruments whose time-varying logarithmic returns differ only by a constant should have the same risk. The advantage of the above rate is already included in its value and not in its volatility. This constant may represent various costs, such as those related to transactions or insurance. These costs do not carry any associated risk. These are known and fixed in advance.*

*By defining such an equivalence relation, we focus on the shape and relative movements of the curves, ignoring absolute position differences.*

## 7. The Nature of Financial Time

When we think about the financial market it is hard to pinpoint a time when it is at a total pause. However, on the other hand the moments of time at which buy–sell transactions take place are rather discrete [23,24]. Many financial events are measured at specific intervals, e.g., portfolio valuations are made at the end of the trading day, dividend payments are made at regular intervals. Most financial data, such as stock prices, interest rates, or macroeconomic indicators, are available in discrete form, i.e., recorded at specific points in time. Discrete-time models naturally fit such a data format, making them easier to use and interpret. Although economic data are rather discrete in nature, econometric models treat them as continuous observations. This approach has a deep theoretical basis, especially in the laws of large numbers or the central limit theorem. The use of continuous models to represent and analyze a variety of economic and investment problems brings unique advantages that make them often preferred over discrete models, especially in the context of complex market analysis and derivative pricing. A key advantage is the ability to use differential calculus, which provides a convenient tool for finding optimal market strategies. It should be mentioned that mixed models, which combine discrete-time and continuous-time elements, are very popular. This hybrid approach allows the advantages of both methods to be exploited and is particularly useful in situations where no single approach can fully capture the complexity of the phenomenon being modeled. The best-known models referring to both continuous and discrete time are the jump diffusion models, where a standard continuous price process, such as Brownian motion, is combined with random jumps to better reflect the actual behavior of prices in the market [25]. Such models allow for a more accurate representation of reality, where asset prices can change both continuously and experience sudden changes as a result of significant events. It is worth noting that discrete models have the advantage that they are often more intuitive and easier to understand.

To determine the risk of financial instruments in discrete time, it is necessary to modify the definitions of a financial instrument graph.

Let us assume that the valuation of a financial instrument is carried out at certain points in astronomical time, starting at time $t_{1}>t_{0}$ and ending at time $t_{n}$. We obtain a finite, ordered sequence of times $t_{1},t_{2},\ldots ,t_{n}$ and $t_{i-1}<t_{i}$ for i=2,3,…,n. As we mentioned earlier, the financial instrument is observed over a certain time interval, hence we assume that the sequence is arbitrarily long but finite. The assumption of the finiteness of this sequence has only practical significance; it remains irrelevant to our model.

**Definition** **2.** *The set*

$$\gamma_{A}\left(t\right):=\left\{\left(\;x\left(t\right),\;y_{A}\left(t\right)\;\right)\;\in R^{2}\;|\;t\in \left\{t_{1},t_{2},\ldots ,t_{n}\right\}\right\}$$

*parametrized by moments of astronomical time t, $t_{i-1}<t_{i}$ for i=2,3,…,n is called a discrete graph of the financial instrument $v_{A}$ on the reference time interval $\left(t_{0},t_{n}\right]$.*

The above definition can be understood as a discretization of a continuous curve (4). This process is often used to simplify the analysis of continuous functions or signals, allowing them to be processed by numerical methods or computer simulations [26,27].

The choice between discrete and continuous time is simply a matter of accepted convention. The ideas presented below can also be used when the graph of a financial instrument is understood as a curve.

## 8. Simple Instruments

In the set of financial instruments, we distinguish the set of **simple instruments** $I_{S}$, i.e., those whose graph in a fixed frame of reference is a line segment:

(5) a·x+b·y+1=0,

where x:=x(t), y:=y(t). In the proposed model, these instruments are characterized by the lowest risk. Later in the article, we provide a precise definition of risk. At this point, it is important to note that while the graph of a risky instrument can be presented either as a set of isolated points (akin to a discrete model) or as a continuous curve, a ’simple instrument’ is always represented by a segment of a straight line. This technical distinction is intended to maintain consistent mathematical formalism, regardless of the graphical representation chosen for a risky financial instrument.

We also consider lines passing through the origin of the coordinate system. We will not discuss them separately because of clarity of considerations, as they do not affect the final results.

In every fixed frame of reference, the most basic examples of simple instruments are the *benchmark instrument* and *money*, whose graphs lie on the lines

y−x=0,y=0,

respectively. This follows directly from the definition of the graph of a financial instrument provided in Section 6.

The model also considers the case of straight lines that decrease in value with respect to the reference time. An illustrative example of this category of instrument is one represented by a straight line with the equation

(6) y+x=0.

The form of the above equation is derived from the observation that if the price of the benchmark instrument $v_{1}$ expressed in money units $v_{0}$ is $U_{t}\left(v_{0},v_{1}\right)$, then the price of money expressed in units of the benchmark instrument is $\frac{1}{U_{t}\left(v_{0},v_{1}\right)}$. This, in light of the property of the logarithm of an inverse number, can be expressed as

$$\ln\frac{1}{U_{t}\left(v_{0},v_{1}\right)}=-\ln U_{t}\left(v_{0},v_{1}\right)\;.$$

The line (6) is symmetrical to the line y−x=0 with respect to x=0. Consequently, the points lying on it represent the logarithmic rates of return on a short position taken on the benchmark instrument and valued in the money $v_{0}$.

A simple instrument can be defined as a pair of numbers, (a,b), representing its coordinates. It is important to note that the characterization of an instrument as simple or not depends on the frame of reference. For instance, the fact that an instrument whose prices are expressed in US dollars is simple does not imply that it is also simple when we change the frame of reference by switching the currency to Japanese yen. This holds true if the relationship between the exchange rates of these currencies is linear.

**Figure 1:**
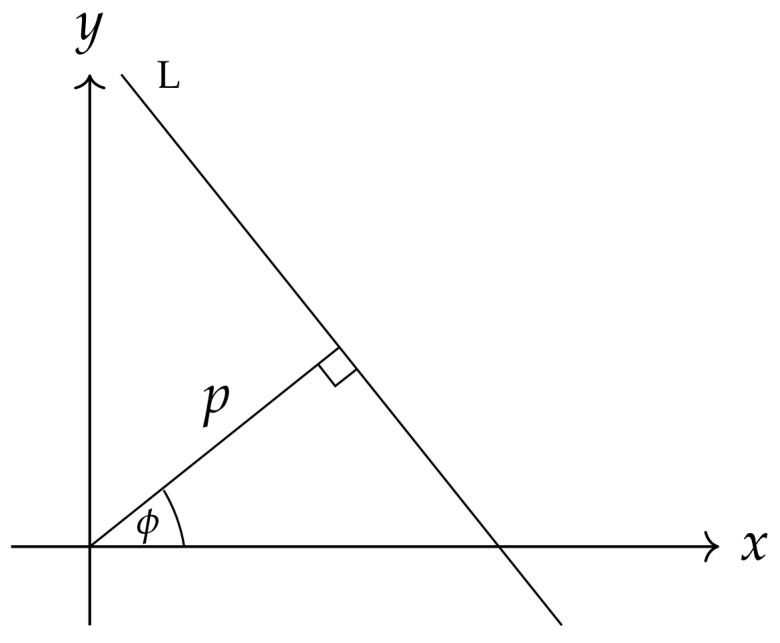
Coordinates to describe a straight line L.

It is convenient to write a simple instrument (a line) in the space $R^{2}$ in the normal form using two coordinates, the directed angle ϕ and the distance *p* of this line from the origin of the coordinate system (Figure 1):

$$\begin{array}{c} p=x\cos(\phi )+y\sin(\phi )\;. \end{array}$$

**Definition** **3.** *The set*

$$I_{S}:=\{\left(\phi ,p\right)\in R^{2}\;|\;\phi \in [0,\pi ],\;p\in R_{+}\}$$

*is called the set of simple instruments.*

In Section 11, we define the *reachability* of risky financial instruments using the concept of simple instruments and the properties of the Dirac delta function and Radon transform.

## 9. The Dirac Delta Function and Its Properties

The Dirac delta function

$$\delta \left(s\right)≃\left\{\begin{array}{cc} +\infty , & s=0\\ 0, & s\neq 0 \end{array}\right.\;$$

is a mathematical concept used to model an infinitely sharp peak at a single point, with the total area under the peak equal to 1:

$$\int_{-\infty }^{\infty }\delta \left(s\right)\;ds=1.$$

It is not a function in the traditional sense but a “generalized function” or “distribution” used in physics and engineering to represent an idealized point mass or charge, and in mathematics to formalize the concept of a point impulse. The Dirac delta function has a number of interesting properties. From the point of view of our considerations, the relevant ones are those given below:

(7) $$\begin{array}{cc} \; & \delta (s)=\delta (-s)\;, \end{array}$$

(8) $$\begin{array}{cc} \; & \delta \left(as+b\right)=\frac{1}{\left|a\right|}\delta \left(s+\frac{b}{a}\right)\;, \end{array}$$

(9) $$\begin{array}{cc} \; & \int_{-\infty }^{\infty }\delta \left(s-a\right)\delta \left(s-b\right)ds=\delta \left(a-b\right)\;. \end{array}$$

## 10. The Radon Transform of a Graph of a Risky Financial Instrument

Let us define the function

(10) $$\begin{array}{c} f_{\gamma_{A}}\left(x,y\right):=\sum_{i=1}^{n}\delta \left(x-x\left(t_{i}\right)\right)\delta \left(y-y_{A}\left(t_{i}\right)\right)\;. \end{array}$$

This is a Dirac delta representation of the graph of a risky financial instrument. The explanation is based on the fact that the expression $\delta \left(x-x\left(t_{i}\right)\right)\delta \left(y-y_{A}\left(t_{i}\right)\right)$ takes the value 0 everywhere except at the point $\left(x\left(t_{i}\right),y_{A}\left(t_{i}\right)\right)$ on the graph of the financial instrument, which follows directly from the definition of the Dirac delta function.

The summation follows all the points of the graph of the risky financial instrument in the interval $\left(t_{0},t_{n}\right]$. If *t* is a continuous parameter, then this representation takes the form of an integral:

$$\begin{array}{c} f_{\gamma_{A}}\left(x,y\right):=\int_{t_{0}}^{t_{n}}\delta \left(x-x\left(t\right)\right)\delta \left(y-y_{A}\left(t\right)\right)\;dt. \end{array}$$

**Definition** **4.** *Let $I_{S}$ denote the set of all simple instruments and ds denote an increment of length along the line of the simple instrument. The function*

$${\overset{ˇ}{f}}_{\gamma_{A}}\left(p,\phi \right)=Rf_{\gamma_{A}}\left(I_{S}\right)=\int_{I_{S}}f_{\gamma_{A}}\left(x,y\right)\;ds,$$

*is called the Radon transform of the financial instrument A.*

**Figure 2:**
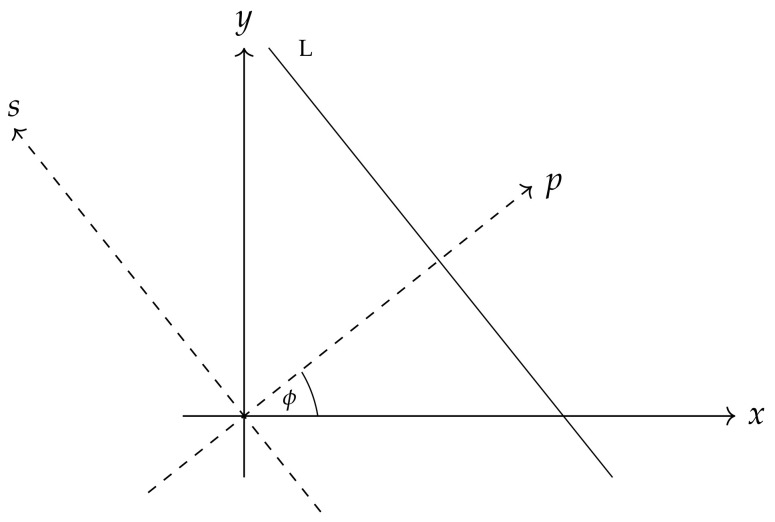
The line from Figure 1 together with the original and rotated coordinate system.

It is useful to introduce a new coordinate system and be somewhat more precise about the integration along all lines from $I_{S}$. The new coordinate system is introduced with axes *x* and *y* rotated by the angle ϕ (Figure 2).

If the new axes are labeled as *s* and *p*, then

$$\begin{array}{cc} x & =p\cos(\phi )-s\sin(\phi )\\ y & =p\sin(\phi )+s\cos(\phi )\;. \end{array}$$

Using the above coordinates, the Radon transform of financial instrument *A* can be written in the following normal form [28]:

$$\begin{array}{c} {\overset{ˇ}{f}}_{\gamma_{A}}\left(p,\phi \right)=\int_{-\infty }^{\infty }f_{\gamma_{A}}\left(p\cos\left(\phi \right)-s\sin\left(\phi \right)\;,p\sin\left(\phi \right)+s\cos\left(\phi \right)\right)\;ds\;. \end{array}$$

To provide an explicit form of the function ${\overset{ˇ}{f}}_{\gamma_{A,\tau }}\left(p,\phi \right)$ let us determine, based on the properties of the Dirac delta, the following integral:

$$\int_{-\infty }^{\infty }\delta \left(p\cos\left(\phi \right)-s\sin\left(\phi \right)-x\left(t_{i}\right)\right)\;\delta \left(p\sin\left(\phi \right)+s\cos\left(\phi \right)-y_{A}\left(t_{i}\right)\right)\;ds.$$

This is the integral of one of the components of the sum (10) for some fixed $t_{i}$. Using the properties (7)–(9), and again (8) successively, we obtain

(11) $$\begin{array}{ccc} & & \int_{-\infty }^{\infty }\delta \left(p\cos\left(\phi \right)-s\sin\left(\phi \right)-x\left(t_{i}\right)\right)\;\delta \left(p\sin\left(\phi \right)+s\cos\left(\phi \right)-y_{A}\left(t_{i}\right)\right)\;ds\\ & & =\delta (p-x\left(t_{i}\right)\cos\left(\phi \right)-y_{A}\left(t_{i}\right)\sin\left(\phi \right))\;. \end{array}$$

**Figure 3:**
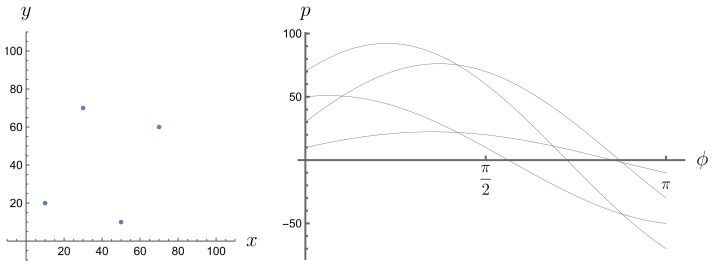
A set of four points, no three of which are collinear, and their Radon transform.

**Figure 4:**
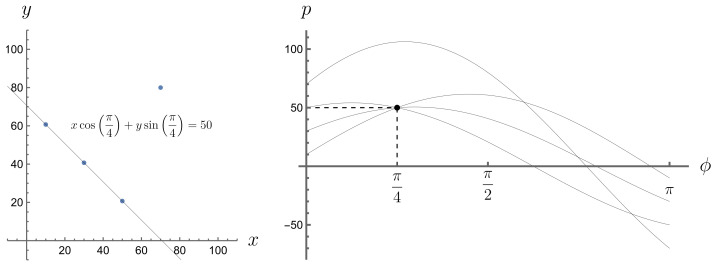
A set of four points, three of which are collinear, and their Radon transform.

**Figure 5:**
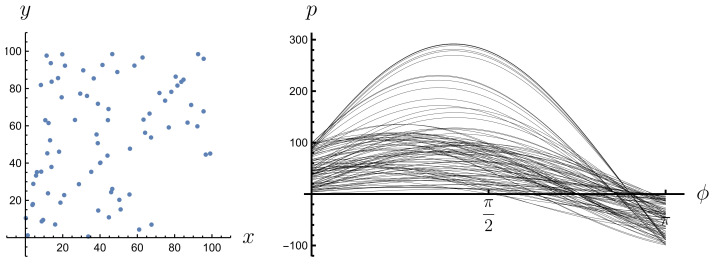
A set of 100 randomly generated points under the condition that at least 45 of them lie on three fixed lines and their Radon transform.

**Figure 6:**
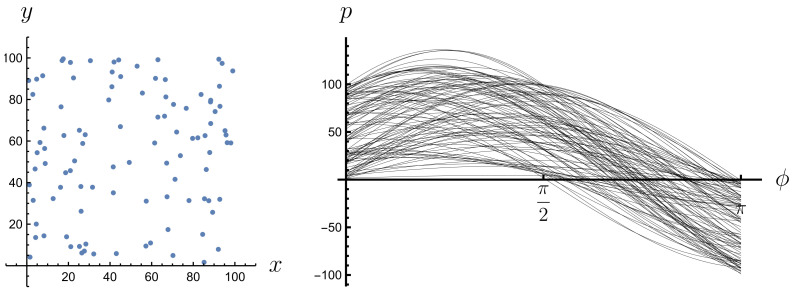
A set of 100 randomly generated points and their Radon transform.

Expression (11) is equal to 0 everywhere except when the δ argument is equal to 0, i.e., when $p=x\left(t_{i}\right)\cos\left(\phi \right)+y_{A}\left(t_{i}\right)\sin\left(\phi \right)$, which means that point $\left(x\left(t_{i}\right),y_{A}\left(t_{i}\right)\right)$ lies on a line of equation p=xcos(ϕ)+ysin(ϕ). The Radon transform of a graph of a risky financial instrument gives us a description of how the points of this graph are distributed in space from the perspective of their projection along different simple instruments. Those simple instruments that pass through more points in the graph of the risky instrument contribute more to the Radon transform than those that pass through fewer points in that graph. This is because they are more frequent in activating the Dirac delta. This effect can be observed in Radon space (ϕ,p); see Figure 3, Figure 4, Figure 5 and Figure 6. A line passing through two or more points is represented in the Radon space by the point of intersection of the sine functions corresponding to these points.

## 11. Reachability and Risk of a Risky Financial Instrument

**Figure 7:**
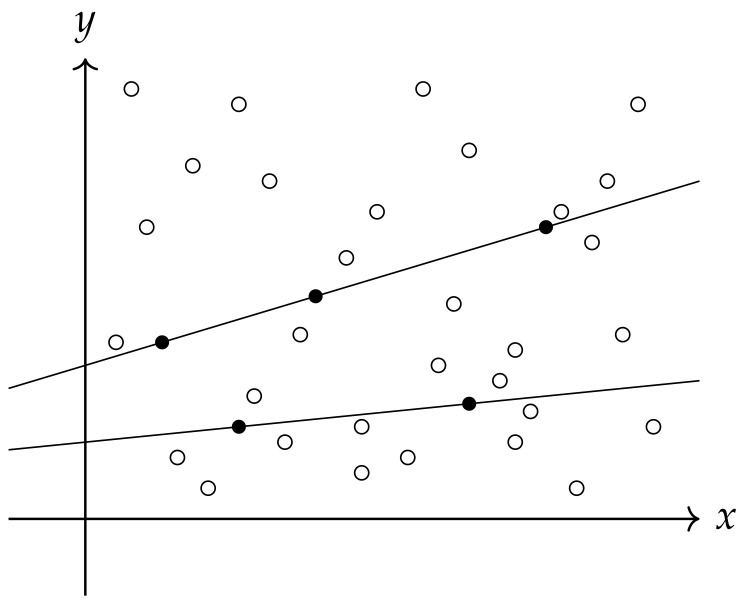
The idea of reachability in a simplified discrete model. The lines represent simple instruments available on a given market. The symbols ∘, • denote historical valuations of a risky instrument in respect to the benchmark instrument. The • marks the moment when a risky financial instrument is reachable.

A risky financial instrument *A* is said to be reachable at a given moment of time *t* if there exists a simple instrument whose logarithmic rate of return at time *t* is equal to the logarithmic rate of return of instrument *A*. This moment can be interpreted as a temptation to sell the instrument if one possesses it, which means to exchange it for a simple instrument, or to purchase it if one does not have it, that is, to replace a simple instrument with instrument *A*. The reachability of a risky instrument *A* within a specific time frame is defined by the number of points of intersection between the graph of instrument *A* and a set of simple instruments. The concept of reachability is illustrated in Figure 7, which depicts the trivial case in discrete time. Our model is general; therefore, the definition of reachability provided below also covers cases where the set of simple instruments has the cardinality of the continuum. However, applying its equivalent in more realistic situations—where the market features a discrete and finite set of simple instruments—poses no problems.

**Definition** **5.** *The quantity*

(12) $$\Delta_{T}\left(A\right):=\int_{0}^{\pi }\int_{0}^{\infty }{\overset{ˇ}{f}}_{\gamma_{A}}\left(p,\phi \right)\;dp\;d\phi$$

*is called the reachability of the financial instrument A in a certain time interval $T=(t_{0},t_{n}]$.*

The Formula (12) counts the intersection points of the graph $\gamma_{A}$ with the set $I_{S}$ during the time interval *T*.

**Definition** **6.** *The risk of financial instrument A is understood as the intensity of relative reachability:*

$${\mathrm{risk}}_{T}\left(A\right):=\frac{\Delta_{T}\left(A\right)-\Delta_{T}\left(I_{A}\right)}{\left|T\right|}\;,$$

*where |T| is the length of the time interval T on which we determine reachability, $I_{A}$ denotes a simple instrument that gives (in the examined time period) the same profit as the instrument A.*

Using the intensity of relative reachability is advantageous as it facilitates comparisons of the risk associated with financial instruments across various time periods. It is important to note that the least risky instrument, typically referred to as the ‘simple instrument’, has a risk value equal to 0, like in the case of measuring the risk of this instrument by standard deviation.

## 12. Why the Intensity of Relative Reachability Is a Natural Measure of Financial Risk

A common and universally accepted measure of financial risk is the standard deviation, an indicator of the volatility of a financial instrument. This measure is based on the extent of price fluctuations of an asset over a given period of time. If its prices tend to fluctuate significantly, there is a greater risk of making the wrong choice. Other risk measures, such as value at risk (VaR) and the systematic risk measure β, also use standard deviation to estimate the volatility and potential impact of external market changes on an investment portfolio. Despite its simplicity, standard deviation can lead to numerous misunderstandings, as highlighted in the intriguingly titled work, “We Don’t Quite Know What We Are Talking About When We Talk About Volatility” by Daniel G. Goldstein and Nassim Nicholas Taleb [29]. The publication reveals how finance professionals often confuse standard deviation with mean absolute deviation, leading to significant underestimations of market volatility.

Standard deviation, although commonly used in risk analysis, only refers to price volatility. It is an objective indicator that can be easily measured and interpreted. However, it does not take into account psychological or social factors that significantly influence investment decisions. Behavioral economics theories, such as prospect theory, show that people perceive risk not only through the lens of volatility but also through a range of other factors, such as risk appetite, past experiences, investment goals, or subjective feelings related to losses and gains. In practice, it is difficult to classify all these factors, and each market participant may perceive the same situation differently, depending on their individual context, which traditional risk measures fail to capture. In our approach, to account for the individual perspective on risk perception, we introduce a frame of reference that allows observing the risk of a financial instrument through the lens of a chosen benchmark instrument and money. The choice of this frame of reference is an individual decision of the investor. The risk measure proposed in this article directly relates to the ability to buy or sell a financial instrument. These abilities are not merely dependent on the technical ability to execute a transaction; they are strongly influenced by individual preferences, shaped by personal experiences and social environment, that is, everything we can call behavioral factors. It seems natural that a number of the dilemmas related to buying and selling would directly affect the assessment of risk.

Any risk measure typically reflects the potential threat faced by the holder of a financial instrument due to the uncertainty of returns. If one of these concerns is the risk of losing the instrument through its sale—for instance, due to pessimistic profitability forecasts—then reachability effectively highlights this threat. The points of intersection of the graph of the financial instrument with a set of simple instruments determine those moments when the logarithmic rate of return on the risky instrument equals the rate on the simple instrument. In such circumstances, a rational investor owning a financial instrument may be tempted to sell it in favor of an instrument with minimal risk, known as a simple instrument. Conversely, if an investor does not possess it, they may be tempted to buy it, exchanging a simple instrument for a risky one. The likelihood of losing an instrument in the future is directly proportional to its potential to be sold within a specified time period. Consequently, this opportunity depends linearly on the reachability of the financial instrument. This measure of the possibility of losing the possessed good can be identified with the risk of possessing it.

It is worth noting that reachability is an extensive parameter of a financial instrument. Doubling the graph of this instrument doubles its reachability. Therefore, in Definition 6, we refer to the ratio of relative reachability to the reference time in which this reachability occurred.

To determine reachability, we use the measure dpdϕ, which is invariant under rotation and translation. It is therefore a measure that remains unchanged with respect to the variation in the rate of return of all simple instruments by any constant, the moment $t_{0}$ at which these instruments are defined, and the global change in the rate of return resulting from shifts in the angle of inclination of the lines to the *x*-axis. Reachability is a measure defined on a set of simple instruments, which are represented by straight lines in a given frame of reference. Therefore, the Radon transform, which converts the curve representing the financial instrument into a set of its projections along each line, seems to be a natural way to describe it.

## 13. Conclusions

The proposed measure of risk for a financial instrument in the paper is closely related to the graph of this instrument. However, there is nothing to prevent considering the graphs of other goods for which we know the time evolution of their utility functions. The concept of reachability can also be easily generalized to graphs in $R^{n}$, which allows for the construction of a consistent theory of risk for complex, multidimensional capital processes.

Astronomical time is a commonly used measure of time lapse in finance. But can it be considered a natural measure? This would be possible if empirical data could demonstrate that market price parameters are periodic in cycles of equal number of hours. However, the observed high and unpredictable volatility in financial markets suggests different conclusions. This leads to the introduction of the concept of reference time. In the proposed model, reference time is described as the definite integral of the instantaneous growth rate of the benchmark instrument. An investor using this concept, when examining the intensity of profit, refers rather to the value of the return rate of the benchmark instrument in a specified astronomical time interval. It is natural for an investor to be more interested in what has happened with the return rate of the benchmark instrument in a specified time interval, rather than the length of that interval itself. The benchmark instrument serves as a reference point for him, against which he values all other financial instruments. Furthermore, reference time can well describe market conditions. The word “recession”, coming from Latin, means moving backward, which in terms of reference time would be expressed by its negative value.

The principle of defining the frame of reference adopted in the paper can explain the mechanisms of differing assessments of the riskiness of the same financial instrument by various market entities. The reachability of a financial instrument is a relative measure because it depends on the arbitrary choice of the frame of reference used to describe the price fluctuations of financial instruments.

The proposed measure can be regarded as a complement to traditional models, adding the element of individual risk perception. This will allow for a more complex analysis that considers not only objective indicators of market volatility but also the subjective perspective of market participants. Such an approach can enhance the accuracy of existing models or inspire the creation of new, more personalized risk assessment tools. A better understanding of the subjective factors influencing financial decisions will enable the identification of common trends in risk perception among different market participants, thereby aiding in the detection of anomalies stemming from collective behavior. For this reason, developing models that account for subjective risk perception can also provide tools for more effective risk management at the macroeconomic level.

As indicated in Section 8, in every frame of reference, the most basic simple instruments are the benchmark instrument and money. Simple instruments are those whose graphs are line segments in the given frame of reference. However, depending on individual preferences, a market participant may, in the proposed risk model, take into account instruments whose graphs are ’approximately’ line segments in the frame of reference, for example, by referring to regression models. Such a graph represents an idealized instrument but sufficiently approximates a real instrument with a certain predetermined level of approximation. As a result, although such an approximate model does not capture every aspect of reality precisely, it is accurate enough for analysis and interpretation in the chosen context, allowing for useful conclusions to be drawn. Models of this type are widely used in science because they allow key features of phenomena to be captured without the need to replicate the full complexity of reality. However, care must be taken when applying them to avoid overestimating their accuracy and to understand the limitations they introduce.

The Radon transform of a selected financial instrument provides information about its reachability from the position of a specified simple instrument with parameters p,ϕ. Averaged uniformly across the entire domain (p,ϕ) over all simple instruments, it introduces a new measure of risk for the selected instrument against the backdrop of the entire market. By using all simple instruments (lines), the Radon transform of the curve representing the financial instrument can be applied, through the inverse operation, to reconstruct this curve. This fully defines the risk. It is worth remembering that the Radon transform is a key tool in the process of image reconstruction in computer tomography, which has revolutionized medical diagnostics [30,31]. In geophysics, the Radon transform is used to analyze seismic data to detect anomalies in underground structures [32]. This tool is successfully applied in image analysis in computer graphics [33], acoustic [34] and radar signal processing [35], character and handwriting recognition [36], as well as in image cryptography [37]. Could it play a similar role in analyzing the dynamics of financial markets? The answer to this question can only be obtained through further research.

In conclusion, it is worth noting that the financial concept of risk is closely related to the concept of measurement error in physics, so well-studied methods of computed tomography can be transferred to the extensive field of error analysis, which is crucial in experimental physics.

## Data Availability

Data is contained within the article.

## References

[1] Kanazawa K., Sueshige T., Takayasu H., Takayasu M. (2018). Derivation of the Boltzmann Equation for Financial Brownian motion: Direct Observation of the Collective Motion of High-Frequency Traders. Phys. Rev. Lett..

[2] Bucci F., Benzaquen M., Lillo F., Bouchaud J.-P. (2019). Crossover from linear to square-root market impact. Phys. Rev. Lett..

[3] McNeil A.J., Frey R., Embrechts P. (2015). Quantitative Risk Management: Concepts, Techniques and Tools.

[4] Aven T. (2012). The risk concept—Historical and recent development trends. Reliab. Eng. Syst. Saf..

[5] Engle R. (2004). Risk and Volatility: Econometric Models and Financial Practice. Am. Econ. Rev..

[6] Borland L. (2002). Option Pricing Formulas Based on a Non-Gaussian Stock Price Model. Phys. Rev. Lett..

[7] Valenti D., Fazio G., Spagnolo B. (2018). Stabilizing effect of volatility in financial markets. Phys. Rev. E.

[8] Mantegna R.N., Stanley H.E. (1999). An Introduction to Econophysics: Correlations and Complexity in Finance.

[9] Sornette D. (2004). Physics and financial economics (1776–2014): Puzzles, Ising and agent-based models. Rep. Prog. Phys..

[10] Raddant M., Matteo T.D. (2023). A look at financial dependencies by means of econophysics and financial economics. J. Econ. Interact. Coord..

[11] Sinha A. (2024). Select Topics of Econophysics.

[12] Makowski M., Piotrowski E.W., Sładkowski J. (2019). Schroedinger type equation for subjective identification of supply and demand. Phys. A Stat. Mech. Its Appl..

[13] Makowski M., Piotrowski E.W. (2022). Transactional interpretation and the generalized Poisson distribution. Entropy.

[14] Makowski M., Piotrowski E.W., Frąckiewicz P., Szopa M. (2021). Transactional interpretation for the principle of minimum Fisher information. Entropy.

[15] Makowski M., Piotrowski E.W., Syska J., Sładkowski J. (2017). Profit intensity and cases of non-compliance with the law of demand/supply. Phys. A Stat. Mech. Its Appl..

[16] Slovic P. (1987). Perception of Risk. Science.

[17] Kahneman D., Tversky A. (1979). Prospect Theory: An Analysis of Decision under Risk. Econometrica.

[18] Han W., Bai B., Raab C., Shum C., Krishen A.S. (2024). Will you choose a low-rating hotel that offers promotions?–Insights from the prospect theory. J. Travel Tour. Mark..

[19] Barberis N.C. (2013). Thirty years of prospect theory in economics: A review and assessment. J. Econ. Perspect..

[20] Kürüm E., Weber G.W., Iyigun C. (2018). Early warning on stock market bubbles via methods of optimization, clustering and inverse problems. Ann. Oper. Res..

[21] Piotrowski E.W., Sładkowski J. (2007). Geometry of financial markets—Towards information theory model of markets. Phys. A Stat. Mech. Its Appl..

[22] Bolster D., Hershberger R.E., Donnelly R.J. (2011). Dynamic similarity, the dimensionless science. Phys. Today.

[23] Capiński M., Kopp E. (2012). Discrete Models of Financial Markets.

[24] Kreps D.M. (2019). The Black–Scholes–Merton Model as an Idealization of Discrete-Time Economies.

[25] Merton R.C. (1976). Option pricing when underlying stock returns are discontinuous. J. Financ. Econ..

[26] Chapra S.C., Canale R.P. (2022). Numerical Methods for Engineers.

[27] Proakis J.G., Manolakis D.K. (2007). Digital Signal Processing: Principles, Algorithms, and Applications.

[28] Deans S.R. (1983). The Radon Transform and Some of Its Applications.

[29] Goldstein D.G., Taleb N.N. (2007). We Don’t Quite Know What We Are Talking About When We Talk About Volatility. http://ssrn.com/abstract=970480.

[30] Barrett H.H., Viergever M.A., Todd-Pokropek A. (1988). Fundamentals of the Radon Transform. Mathematics and Computer Science in Medical Imaging.

[31] Prestini E. (2016). The Radon Transform and Computerized Tomography. The Evolution of Applied Harmonic Analysis.

[32] Thorson J.R., Claerbout J.F. (1985). Application of Radon Transform to Seismic Data. Geophysics.

[33] Chen C.-H., Lin S.-F. (2009). Radon Transform for Digital Image Processing. J. Vis. Commun. Image Represent..

[34] Beylkin G. (1987). Discrete Radon Transform. IEEE Trans. Acoust. Speech Signal Process..

[35] Ming Y., Jin S. (2018). Radar Signal Processing Using Radon Transform. IEEE Trans. Geosci. Remote Sens..

[36] Kittler J., Illingworth J. (1987). The Use of Radon Transform in Character Recognition. Int. J. Pattern Recognit. Artif. Intell..

[37] Li H., Xie X., Zhang Z. (2012). Application of Radon Transform in Image Cryptography. J. Comput. Appl. Math..

